# Strategic Assessment of Fisheries Independent Monitoring Programs in the Gulf of Mexico

**DOI:** 10.1371/journal.pone.0120929

**Published:** 2015-04-02

**Authors:** Paul Mark Suprenand, Michael Drexler, David L. Jones, Cameron H. Ainsworth

**Affiliations:** 1 University of South Florida—College of Marine Science, 140 7^th^ Ave S, St. Petersburg, Florida, 33701, United States of America; 2 Mote Marine Laboratory, 1600 Ken Thompson Parkway, Sarasota, Florida, 34236, United States of America; Dauphin Island Sea Lab, UNITED STATES

## Abstract

This study evaluates information produced from 14 fisheries independent monitoring programs (FIM) in the Gulf of Mexico. We consider the uniqueness of information from each program and its usefulness in estimating fisheries management indices. Biomass values of 35 functional groups are extracted from an operating model (Ecospace) with a method that replicates the patterns of historic FIM samplings. Observation error is added to these data in order to create a set of pseudo data that replicate the type and quality of information obtained from FIM programs. The pseudo data were put into a separate fishery assessment model (Pella-Tomlinson) to determine management indices of each functional group (maximum sustainable yield (MSY), biomass at MSY, and fishing mortality at MSY). These indices are compared against values in Ecospace, and against previously published single-species stock assessments. We also evaluate the full suite of information derived from FIM within an ecosystem context, considering whether functional roles are over- or under-sampled, and whether sampling effort is proportional to the value of fish stocks. Results reveal that model derived fishery indices closely matched published indices for the majority of the functional groups, economic and ecological evaluation suggests that several piscivorous functional groups are under-sampled include forage base species that are likely to indirectly support fisheries for piscivores, and sampling efforts are not proportional to the value of some fish stocks. Following ecological modelling we performed statistical analyses on historic FIM catch data to identify optimal species-specific sampling months and gear-types that can be used to refine future FIM sampling efforts.

## Introduction

Data yielded from fisheries independent monitoring (FIM) programs in the Gulf of Mexico (GOM) provide the basic information for federal and state stock assessments on numerous exploited species. Stock assessments consider species-specific information from FIM data obtained by federal agencies such as the National Oceanographic and Atmospheric Administration (NOAA) or state agencies such as the Florida Fish and Wildlife Research Institute (FWRI). The data collected typically includes numbers or weights caught, taxonomy, morphometrics, as well as the dates and locations of the capture events. These agencies utilize several fishing gear-types to sample the marine ecosystems, such as shrimp trawls, traps and long-lines. Ultimately, stock assessments based on FIM sampling data, allow the setting of safe harvest limits (e.g., [1]). Unless we feed accurate and representative stock information into the stock assessments, management decisions could lead to harvest levels that are too low, threatening viability of the fishing industry, or too high threatening the marine ecosystem (e.g., [2–7]).

Typically FIM programs are developed and managed independently and usually on a state-by-state basis. As a result they are not optimized for data collection across the ecosystem as a whole, and they may overlap in species, age classes, depths or habitats sampled (providing redundant information). FIM sampling methods may under-represent certain species, or they may allocate sampling effort disproportionately to the commercial or recreational importance of a species. Moreover, areas sampled may be more or less useful for the stock assessment of certain species when considering native distributions and habitat usages.

More recently fishery assessments have begun using whole-ecosystem models to develop management strategies in other parts of the world, as these models describe important trophodynamics missed in single species assessments [8–10]. In general, ecosystem models have been applied to support single species assessments. Whole-ecosystem models have the ability to not only quantify ecosystem connectivity (predator-prey interactions), they can also test maximum fishing mortality scenarios per species with resulting whole-ecosystem responses, the creation of seasonal or geographic fishing closures to estimate overfished species-specific recovery times, or even environmental drivers of migration [11–15]. There are even higher resolution ecosystem models that capture fine-scale details of time [16] and biogeochemistry [17] for better predictions of plankton dynamics when considering physical oceanography and various influences to the marine ecosystem (e.g., water mass advection, light and/or nutrients).

Combining recent advancements in ecological modelling with FIM data has the potential to be an effective tool for more accurately describing important trophodynamic links in the marine ecosystem, as a single species distributions, biomass and fishing limits are affected by environmental and ecological connections [18–20]. However, in the GOM there are only a few published ecosystem models, and those models they have been limited to estimating ages [21], levels of natural mortality [22], or population connectivity [23–26]. While improved estimates of these parameters for stock assessment models are important, accurate fishery independent indices of abundance remain critical for providing effective evaluations of a stock’s status [e.g., 27].

Fishery managers and marine scientists currently recognize the need to expand FIM sampling programs for more effective management of fishery resources [28], as more focused sampling efforts could provide a continuous baseline of data on stock status. Although FIM is naturally limited by the selectivity of sampling gears or the accessibility of certain habitats, no previous study in the GOM has considered the resulting value of FIM information from a whole-ecosystem perspective, nor assessed how sampling can be improved to yield more representative data for the entire suite of stock assessments. Using a whole-ecosystem model can help focus FIM sampling efforts, maximizing scientific benefit across a range of managed species, and also allows for the comparison of fisheries assessments based on target species (historic) versus whole ecosystem; both of which can be used for more adaptive fisheries management indices [29,30].

The current study quantifies the information value of various sampling programs within the Southeast Area Monitoring and Assessment Program (SEAMAP; [31]) and the Comparative Assessment of Gulf Estuarine Systems (CAGES; [32])) using spatial ecological modelling, FIM data, and fisheries assessments. We propose which components of individual programs, if expanded, would yield the greatest improvements in the accuracy of stock assessments. To accomplish this we develop a spatial operating model. We treat the operating model as the “true” ecosystem, then species’ biomass values, catch values, ratios, and fisheries management indices throughout the GOM become ‘known values’. We sample biomass from the operating model at the same locations as the actual FIM programs and with the same level of accuracy by accounting for observational error, and this data is used to estimate common fisheries management indices (e.g. maximum sustainable yield (MSY), biomass at MSY (B_MSY_), and fisheries mortality at MSY (F_MSY_)) with an assessment model. Fishery indices generated by the operating and assessment models are then compared to one another, as well as to historic, current and suggested fishing mortality rates including F_MSY_, the maximum fishing mortality threshold, and other management indices for the GOM. By assessing the accuracy of the indices derived from the assessment model relative to the known values of the operating model, we get an idea of the accuracy of each stock assessment, and therefore the information content of the FIM data on which it relies. Additionally, we evaluated the raw historic FIM catch data using multivariate analysis to determine which sampling years and FIM program characteristics contributed the most information by species. Specifically, we identify best sampling habitats, months and gear-types for obtaining data on many species in the SEAMAP and CAGES programs with the goal of maximizing total scientific value from future FIM sampling.

## Methods and Materials

### Operating Model

**Fig 1:**
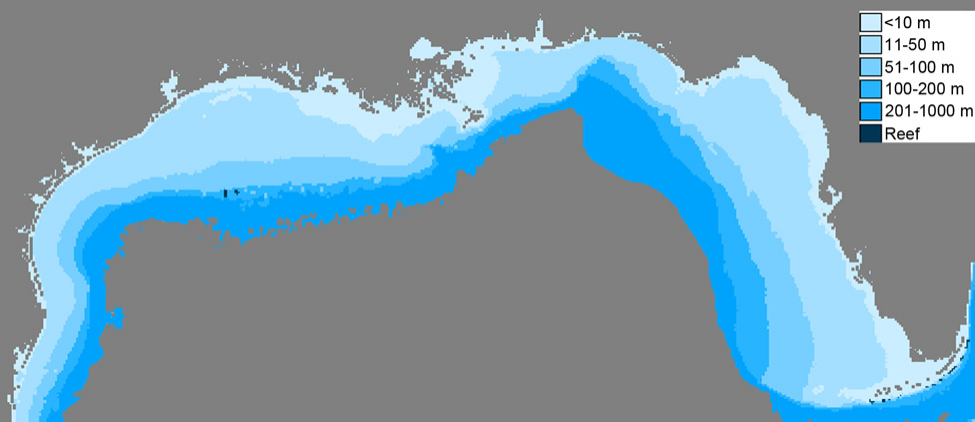
High resolution Ecospace map ranging from 24 to 31°N latitude and 80 to 98°W longitude, and a 4.4 km x 4.4 km resolution per map cell. Depths greater than 1000 m in the central GOM and Florida’s east coast are omitted from ecological analyses.

We developed a spatial operating model of the northern GOM based on Ecospace [33]. The model domain ranges from 24–31°N latitude to 80–98°W longitude, including the coastlines and estuaries from Texas to the Florida Keys, and depths from 0–1000 m (Fig 1). The model was based on an Ecopath with Ecosim (EwE) model [34], which considered 48 functional groups, including single species, aggregated groups of species, and age-structured species. Our amendments to the model include the addition of seven defined habitat types with functional group habitat preferences, the addition of specific Marine Protected Areas (MPAs) to restrict fishing effort spatially, and simulated input of annual chlorophyll *a* concentrations to influence basal trophic energetic exchanges.

The Ecopath with Ecosim model organizes individual species, multi-stanza (or multiple age classes) species, and aggregated groups of species into functional groups. The model acts like a thermodynamic accounting system, tracking material flows through the ecosystem and functional groups according to the constraints of mass-balance and conservation of energy. Ecopath represents an instantaneous ‘snap-shot’ of material fluxes in the ecosystem (see [35] for equations), Ecosim adds a temporal dimension, predicting biomass change for primary producers and consumers according to Eqs 1 and 2, respectively, and Ecospace adds a spatial dimension based on Ecosim simulations in each pixel, adjacent pixels, and habitat preferences (discussed below).

$$\frac{dB_{i}}{dt}=cB_{i}{\left(\frac{P}{B}\right)}_{i}EE_{i}-\sum_{j=1}^{n}f\left(B_{i},B_{j}\right)-M_{i}B_{i}$$ (1)

$$\frac{dB_{i}}{dt}=cg_{i}\sum_{j=1}^{n}f\left(B_{j},B_{i}\right)-\sum_{j=1}^{n}f\left(B_{i},B_{j}\right)+I_{i}-B_{i}\left(M_{i}+F_{i}+E_{i}\right)$$ (2)

*B*_*i*_ and *B*_*j*_ are biomasses of prey (*i*) and predator (*j*), *P* is production rate, *EE* is ecotrophic efficiency, *f* is a relationship predicting consumption, *I* is immigration, *M* and *F* are natural and fishing mortality, *E* is emigration, *g* is growth efficiency, and *n* is the number of functional groups. The scalar *c* is used in this article to introduce forcing functions on productivity (described below), and *EE* is the proportion of the production used in the marine ecosystem.

The operating model (Ecospace) map is comprised of a grid of pixels or cells that each represents an individual Ecosim simulation and habitat type. Each functional group in the model is assigned a set of habitat preferences. Each map cell, with the exception of land cells, thus predicts biomass densities of multiple species and age classes, predator-prey interactions, and fishing mortalities based on trophodynamics, which affects adjacent cells and spatial distributions. Six of the habitat types are created to describe the depth ranges that are <10 m, 11–50 m, 51–100 m, 101–200 m, 201–1000 m, and > 1000 m according the previous EwE model [34]. Depths greater than 1000 m and Florida’s east coast are not considered in the Ecospace model as this study focuses primarily on coastal functional groups in the GOM, and the majority of fishing activities and FIM sampling efforts took place in shelf waters. A seventh habitat type, “Reef,” is created using spatially referenced reef point data downloaded from the ReefBase website (http://www.reefbase.org; [36]). Reefbase aggregates information from scientific studies and crowd sourced data pertaining to coral reefs with the goal of producing information to support decision making by fisheries and environmental managers.

Gulf of Mexico habitat types based on depth ranges are determined from a bathymetry map derived from the SRTMS_PLUS V6.0 data files made available by the Shuttle Radar Topography Mission (SRTM; [37]). The SRTM data was collected using radar interferometry [38], and has a resolution of approximately 1.85 km. A grid of points each separated by 1/25th of a decimal degree was overlaid upon the SRTM bathymetry map as well as the spatially referenced reef point data in ArcGIS 10.0 [39] to create the operating model map with a resolution of 4.4 km x 4.4 km per cell. The cell resolution was chosen based on the average distances between FIM sample collection sites within the Gulf of Mexico, which captured the start and end latitudes and longitudes of FIM trawling efforts (e.g. shrimp trawls), or individual latitude and longitude points of a stationary FIM sample point (e.g. trap or camera recording). This point grid is also used to translate the locations of marine protected areas (MPAs) and values of chlorophyll *a* to operating model cells using the data sources described below.

The operating model domain includes spatially referenced MPAs based on a list of 21 MPA spatial fisheries closures. These help to apportion fishing effort realistically in the spatial model. The dates enacted, names, and respective restrictions are detailed in the online report at the University of South Florida [40]. These 21 MPAs were chosen as they were the most restrictive in terms of limiting fishing activities. When data pertaining to fisheries restrictions were not included in the database of the National Marine Protected Areas Center [41], a basic internet search was performed. A typical search for supplemental data included official local, state, and national park websites, fisheries management agency websites, Google scholar searches by MPA name, as well as fishing and travel blogs. Only a small portion of GOM MPA’s had specific restrictions on fishing. For example, areas such as national wildlife refuges, and state and local parks did not have any specific restrictions beyond their respective state and national regulations despite their listing in the database. MPA’s with site-specific management plans that may restrict fishing in a few small localities are not considered due to their scale.

Chlorophyll *a* concentrations in the model domain were determined according to remotely sensed data downloaded from the Giovanni online data system (Giovanni MODIS-Aqua data) maintained by NASA GES DISC at http://giovanni.gsfc.nasa.gov [42]. Giovanni MODIS-Aqua data at a 4 km resolution was collected from Ocean Portal’s Ocean Color Radiometry Online Visualization and Analysis’ global monthly products, and then averaged across this study’s sampling region by annual concentrations from 1982 to 2012. For a MODIS-Aqua data point that correlated to an Ecospace cell that had no chlorophyll *a* data, two-dimensional linear interpolation was performed using Delaunay triangulation of the scattered data [43,44]. Average annual chlorophyll *a* concentrations were translated into map cells using the point grid system.

### Extracting Simulated FIM Data from Operating Model

Of the 48 functional groups driving trophic energetic exchanges in the Ecospace model, we narrowed our analytical focus to 35 functional groups and their species that are considered in state and/or federal fishing regulations, and/or had reef-related, pelagic and demersal species (Table 1). We then extracted biomass (tonnes km^-2^) and catch (tonnes km^-2^ y^-1^) from a 30-year Ecospace simulation for each of the 35 functional groups by replicating the yearly sampling patterns of FIM programs. This was achieved by converting reported FIM sampling locations (latitudes and longitudes) where each of the 35 functional groups were captured during a given year into an equivalent matrix of Ecospace cells from which to extract biomass and catch data. FIM sampling matrices, or geographically defined pixels of across the Gulf of Mexico, describe the sampling locations for each year in the SEAMAP program (2000 to 2012), and the CAGES program (2000 to 2007). Each sampling matrix therefore characterizes the “year-pattern,” or the total of the unique sampling locations in the operating model’s cells, as reported in an FIM database for that year. By using individual year-patterns to extract the total biomass and catch from the operating output files (files that summarize the biomass/catch per cell per species and per year) we replicate FIM program’s sampling efforts for a given year in terms of sampling areas (i.e. habitats/depths), species and age classes sampled. Therefore, the depths, habitats, species and age classes accessible by all gear-types involved in FIM sampling are realistic and informed by the historical FIM data.

**Table 1:** Species comprising the 35 functional groups in the 14 FIM programs considered in analyses in the present study.

Functional Group	Species	Functional Group	Species
**Atlantic Croaker**	*Micropogonias undulatus*	**Lobster**	*Nephropsis spp*.
**Bay Anchovy**	*Anchoa mitchilli*		*Nephropsis aculeata*
**Blue Crab**	*Callinectes spp*.		*Nephropsis rosea*
	*Callinectes sapidus*		*Polycheles spp*.
	*Callinectes similis*		*Polycheles typhlops*
	*Callinectes ornatus*		*Panulirus argus*
**Catfish**	*Arius felis*		*Scyllaridae spp*.
	*Bagre marinus*		*Scyllarides aequinoctialis*
	*Ictalurus furcatus*		*Scyllarides delfosi*
	*Ictalurus punctatus*		*Scyllarides depressus*
**Grouper**	*Epinephelus spp*.		*Scyllarides nodifer*
(ages 0–1, 1–3, >3)	*Epinephelus adscensionis*		*Scyllarus spp*.
	*Epinephelus drummondhayi*		*Scyllarus americanus*
	*Epinephelus flavolimbatus*		*Scyllarus chacei*
	*Epinephelus guttatus*	**Mackerel**	*Auxis rochei*
	*Epinephelus itajara*	(ages 0–3, >3)	*Scomber japonicus*
	*Epinephelus morio*		*Scomber colias*
	*Epinephelus nigritus*		*Scomber scombrus*
	*Epinephelus niveatus*		*Scomberomorus cavalla*
	*Mycteroperca bonaci*		*Scomberomorus maculatus*
	*Mycteroperca interstitialis*		*Scomberomorus regalis*
	*Mycteroperca microlepis*	**Menhaden**	*Brevoortia patronus*
	*Mycteroperca phenax*	(Juveniles &	*Brevoortia gunteri*
	*Mycteroperca venenosa*	Adults)	*Brevoortia smithi*
**Jacks**	*Caranx spp*.	**Mullet**	*Mugil cephalus*
	*Caranx crysos*	(ages 0–6, 6–18, >18)	*Mugil curema*
	*Caranx hippos*	**Pigfish**	*Orthopristis chrysopterus*
	*Hemicranx amblyrhynchus*	**Pin Fish**	*Diplodus holbrooki*
	*Seriola spp*.		*Lagodon rhomboides*
	*Seriola dumerili*	**Pompano**	*Alectic ciliaris*
	*Seriola fasciata*		*Rachycentron canadum*
	*Seriola rivoliana*		*Trachinotus carolinus*
	*Seriola zonata*		*Trachinotus falcatus*
**Ladyfish**	*Elops saurus*	**Red Drum**	*Sciaenops ocellata*
(ages 0–10, >10)		(ages 0–3, 3–8, 8–18,	
**Lobster**	*Homarus*	8–36, >36)	
	*Munida spp*.	**Red Snapper**	*Lutjanus campechanus*
	*Munida flinti*	(ages 0–6, 6–24, >24)	
	*Munida forceps*	**Scaled Sardine**	*Harengula jaguana*
	*Munida iris*	**Sea Trout**	*Cynoscion spp*.
	*Munida irrasa*	(ages 0–3, 3–18, >18)	*Cynoscion arenarius*
	*Munida longipes*		*Cynoscion nebulosus*
	*Munida pusilla*		*Cynoscion nothus*
	*Munida robusta*	**Silver Perch**	*Bairdiella chrysoura*
	*Munida simplex*	**Stone Crab**	*Menippea spp*.
	*Munida valida*		*Menippe adina*
	*Munidopsis spp*.		*Menippe mercenaria*

Not all species considered were present in the 14 FIM programs. Ages are in years.

Using this method we analyzed 14 FIM programs. Thirteen programs were described based on the gear-types used in the SEAMAP program, which included gear-type Bib Trawl (BB), Bottom Longline (BL), Experimental Shrimp Trawl (ES), Fish Trawl (FT), Hand Line (HL), High Opening Bottom Trawl (HO), Off-bottom Longline (OB), General Plankton (PN), Standard Mongoose Trawl (SM), Shrimp or SEAMAP Trawl (ST), Fish Trap (TR), Video Trap (TV), and the Video Camera (VC). The fourteenth FIM program was the estuarine sampling from CAGES. The database does not specify gear-type used. All 13 SEAMAP programs’ reported in the historic catch records were used to construct each SEAMAP year-pattern, and the CAGES program, describing the sampling of all 5 Gulf States, were used to construct each CAGES year-pattern.

This method creates a set of ‘pseudo’ sampling data that represents the same kinds and quality of information that are obtained from FIM programs. Just as fisheries managers would do in real life, we use this pseudo data in a separate assessment model to estimate common fisheries management indices (maximum sustainable yield (MSY), biomass at MSY (B_MSY_) and fisheries mortality at MSY (F_MSY_)). We have assumed that a sampling event located within an Ecospace cell would return a biomass density representative of that cell. This is not such a strong assumption, as our cells are only 4.4 km by 4.4 km and are designed to represent homogeneous habitats. In fact, a similar assumption is implicitly present in any sampling program that extrapolates swept-area measurements up to the scale of the whole stock or occupied range. Obviously, FIM programs sample in different areas each year and with different amounts of total sampling effort using an intentional random sampling strategy. This variability gives us the opportunity to examine which areas provide the most valuable information for stock assessment on a species-by-species basis.

Obviously, FIM programs sample in different areas each year and with different amounts of total sampling effort using an intentional random sampling strategy. This variability gives us the opportunity to examine which areas provide the most valuable information for stock assessment on a species-by-species basis.

With the total biomass within sampled cells extracted from Ecospace and summarized by year-pattern and FIM program, biomass is scaled to represent the total biomass of each functional group (or species) over the entire operating model domain using a swept-area method. For each functional group, total system biomass (*B*) was estimated by dividing the sample area biomass values (*b*) in each sampled cell (*j*) by the number of unique Ecospace cells sampled by that year-pattern (*n*), and then multiplying the answer by the number of Ecospace cells (*S*) inhabited by that specific functional group. (Eq 3 below)

$$B\;=\;S∙\sum_{j\;=\;1}^{n}\frac{b_{j}}{n}$$ (3)

For any functional group, a cell was considered inhabited if its biomass value was greater than 10% of that group’s maximum biomass density. This was stipulated because of the Ecospace diffusion algorithms which yield functional group biomass values in almost all Ecospace cells. A cell that has greater than 10% of the functional group’s maximum biomass density thus allows for the determination of the optimal habitat(s) for each functional group based on trophodynamics, and 9 out of every 10 cells are consequently inhabited in each group’s habitat type. Inhabited cell biomass values extracted from the operating model represented the stable equilibrium condition and so were sampled after transient dynamics calmed in the model, usually after year 10. This method estimated the total biomass in the GOM marine ecosystem for each functional group and year based on year-pattern sampling efforts in FIM programs.

As there would be error associated with FIM sampling in the GOM, the estimated final “observed biomass” values (per FIM program year-pattern and species) are determined by adding an appropriate amount of observational error. Observational error is calculated using the SEAMAP database. Normal observational error is added to the operating model’s extracted biomass values such that the standard deviation matches that observed in SEAMAP data when averaged over all species and assessed within a five-year running mean (~16%) in order to exclude variance from directional change and consider only year-to-year variability. SEAMAP data is used for this calculation as it reported total kg of animals captured per sampling effort, whereas CAGES reported only the number of animals captured per sampling effort [45]. Overall it is difficult to estimate observational error in trawl sampling without a dedicated study. A minimum estimate of the observational error might be made by looking at variance of population estimates over short time periods (i.e. short enough periods that any changes in the population size can be assumed negligible). However, estimating variance over longer time periods (i.e. over time scales at which population changes are likely to occur) is one way of placing an upper limit on the amount of observational error that is possible. We compared the variance in biomass estimates over short and long periods to develop an estimate of the likely range of observational error. Variance in biomass ranged from 13.4% for a 3-year running average to 16.5% for an 11-year running average. We elected to use a coefficient of variation of 16%, representing the average variability over a 10-year moving window. That is long enough that our estimate of observational error may be inflated by population size changes, but we consider it safer to over-estimate observational error than underestimate it. In fact, the more salient concern is our assumption that the same degree of observational error can be assumed for all sampling gear types. Since the data record for those other gear types is non-continuous, and since no previous studies are available estimating observational error for those gear-types, we assume as a first estimate that the observational error from trawl is similar to that of other gear types. In fact, more than 94% of the data used by our assessment model was produced by Shrimp Trawls, so this assumption is reasonable. The resulting final observed biomass and catch estimates (pseudo data) are used in the assessment model.

### Assessment Model

A Pella-Tomlinson stock assessment model [46] is fit to the pseudo data biomass values and operating model-wide catch data from fisheries statistics. The catch data are assumed to be free of error, since this information is comparatively precise in real stock assessments. An assessment is made for each year-pattern and functional group. The Pella-Tomlinson model contains three parameters, which are varied in order to maximize log likelihood using the Solver function in Microsoft Excel. The first parameter is the theoretical Biomass at infinity (B_inf_), the second is the intrinsic rate of population increase (r; a parameter that captures aspects of mortality, reproduction, and tissue growth), and the third parameter is the shape (p), which was allowed to vary between 0.0001 (approximating a Schaeffer model; [47]) and 1.0 (approximating a Fox model; [48]). Log likelihood compares the goodness of fit of the observed Ecospace biomass values and the predicted assessment model biomass values. With the model fitted to data, the fishery management indices are determined for each functional group and year-pattern sampling effort. For the multi-stanza functional groups (groups with multiple age classes), aggregated biomass and catch values were inputted into stock assessments.

The F_MSY_ values generated by the Pella-Tomlinson stock assessment model for each functional group are compared to the F_MSY_ values calculated by incrementally changing fishing mortalities in Ecospace (i.e. an equilibrium analysis). This is accomplished by increasing fishing mortality for all fished functional groups simultaneously, starting with fishing mortalities (F) of 0.0 times the baseline Ecopath F and then incrementally increasing F for all functional groups by 0.1 until it reaches a value of 29.0 times the baseline Ecopath F. A value of 29.0 times the baseline Ecopath F was needed to ensure that the majority of exploited groups collapsed. This method yields operating model estimates of each functional group’s maximum fishing mortality for F_MSY_. Note that this manual technique is required because Ecosim’s equilibrium fitting routine does not operate in Ecospace; the equilibrium fitting routine would be inadequate for multi-stanza groups as the F of only a single age class can be incremented.

### Analyses of Operating-Assessment Model Agreement

To quantify variability explained by the proportion of Ecospace habitat sampled in each year-pattern, we use the operating and assessment model residuals of MSY, B_MSY_, and F_MSY_ as dependent variables Redundancy analysis (RDA; [43,49–51]) was used. RDA tests performed forward-stepwise selections of the Ecospace habitats that included forward addition of those variables based on the Akaike’s information criterion (AIC). AIC was used to estimate Kullback-Leibler information loss by having a 'lack-of-fit' term and a number of parameters penalty for statistical model complexity. RDA with AIC was used to test all functional groups concurrently, and then run to detect individual functional group results. The results of the RDA tests lead to the selection of the optimal subset of habitat types that explained the majority of variability in residuals of MSY, B_MSY_, and F_MSY_, and corresponding R^2^ values were reported. This method of RDA was free of the reliance of F-ratios (F) and P-values (P) to determine significance [52]. By assessing the accuracy of the indices derived from the assessment model relative to the known values of the operating model, we get an idea of the information content of the FIM data. Moreover, we can describe the utility of each FIM program as it contributes to the stock assessment of managed species. For example, FIM programs that sample on the edge of a population, in marginal habitats or depth ranges, or catch a limited subset of age classes may yield biomass information that is not representative of the overall population status. Alternatively, overlap by two or more FIM programs in the depths, habitats, species, or age classes sampled, may indicate redundancy in FIM sampling effort.

To determine which functional groups were over- or under-sampled in the SEAMAP program in relation to the size of their fishery, a figure was created by plotting the mean discrepancy (coefficient of variations estimated between the assessment and operating model) of MSY against Ecopath catch values [34]. Similarly, the mean discrepancy in MSY are plotted against functional groups’ catch value and keystoneness to give an idea of sampling effort versus economic and ecological importance of functional groups [53,54]. Keystoneness is a measure of how much changes in the groups’ biomasses affect the rest of the ecosystem; a keystone species has an effect disproportionate to its biomass. The protection of keystone species helps ensure the function of the ecosystem and its natural resources, therefore keystoneness was included. In each of the three plots (i.e. MSY coefficients of variations plotted against Ecopath catch, catch value, and keystoneness) regression lines were added to illustrate trends in operating and assessment model agreements. Significance of the regression line slopes (i.e. whether there was a true relationship between X and Y) were assessed using permutation-based t-tests (iterations n = 1000; [51]) for testing the null hypothesis that the slope of the regression was not significantly different than zero. If two regression lines were plotted within a single figure the equivalence of slope parameters were tested to determine whether or not the two regression lines (trends in operating and assessment model agreements) were significantly different from one another using permutation-based t-tests (iterations n = 1000). Following all permutation based t-tests P-values are reported, and significance is concluded if a resulting P-value is <0.05.

### Statistical Analysis of Raw FIM Catch Data

We calculated the average long-term ratios of relative abundance (catch per unit effort, CPUE) per species from the FIM data. Although CPUEs are expected to vary from year-to-year, the gross ratio across all species may be constrained by total system productivity and trophic transfer efficiencies. For SEAMAP we averaged the species’ CPUE ratios from 2000 to 2012 and for CAGES we calculated the species’ CPUE ratios from 2000 to 2007. To see which year-pattern(s) produced the most representative estimates of long-term CPUE by species, we conducted a RDA in which years (e.g. 2000, 2001…2012 for SEAMAP data) were the independent variables, and CPUE was the dependent variable. The years that best explained variability in CPUE were reported with corresponding R^2^ values.

We then focused on testing two null hypotheses regarding the sampling characteristics in the FIM programs: 1) species caught by FIM surveys show no significant differences in relative abundances when analyzed with respect to the month of sampling, and 2) species caught showed no significant differences in relative abundances with respect to the gear-types used for sampling (SEAMAP database only). To test for significant differences in species abundances by month or gear-type, a non-parametric multi-way analysis (np-Manova; [51,55]) evaluated these hypotheses with distribution-free randomization tests (n = 1000 iterations). Null hypothesis testing required the creation of dependent variable matrices based on Bray-Curtis dissimilarity. One matrix was comprised of all species’ relative abundances reported in the SEAMAP database [31], and four more matrices were created for each Gulf State and their species found in the CAGES database [32]. Significance is concluded if a resulting P-value is <0.05, and the null hypothesis is rejected. The F-ratios and the number of observations (N) considered in each np-Manova test are reported.

Following a significant result in an np-Manova test, a canonical analysis of principal coordinates (CAP) test was used to detect the fraction of variability in abundance that was explained by the category (i.e. month or gear-type) considered [43]. CAP uses a non-parametric Canonical Discriminant Analysis that utilizes a Bray-Curtis dissimilarity matrix [44,56–58]. This matrix was created using abundance data according to the category in the null hypothesis being tested. Each CAP test yielded the proportion of within group variation and percent of reclassification success in the abundance data by category, and following 1000 iterations, provided a P-value [43,51,58,59].

To establish if the percent reclassification success was significantly different than the null model that assigned categorization of abundance data merely by chance, a Proportional Chance Criterion (PCC) test was applied following the CAP test [43,51]. The PCC test described the success of categorizing abundance data merely by chance, and generated a P-value that indicated whether or not the fraction variability reported in the CAP test was significantly different than the percent variability reported in the PCC [60,61].

To identify those species that contributed to the most differences detected in an np-Manova and CAP test, the abundance data were then analyzed using RDA with AIC, and the methods to identify indicator species [62]. The results of RDA with AIC lead to the selection of the optimal subset of categorical variables and the identified indicator species that were characteristic of a particular categorical variable or variables (i.e. specific month(s) or gear-type(s)) based on the categorical affinity value. An indicator species characteristic of one categorical variable had a higher categorical affinity value (e.g. 100%), which indicated that it occurs in one category and in all samples within that category. A species characteristic of two or more categorical variables would subsequently have a lower affinity value, as the total percent (affinity) would be shared between categories. The categorical affinity value, henceforth referred to as the Indicator Value (IV), ranges from 0 to 100%, and affinity is maximum (100%) when a functional group or species occurred in all samples of that categorical variable. Thus, species that a particular month or gear-type is exceptional at sampling are identified as having a high IV. These months and/or gear-types are therefore potentially irreplaceable in the assessment of these species, as they are essential for sampling of the species. The IV value and associated P-value were reported for species with IV greater than a ten-percent association with the categorical variable. As the CAGES database did not specify gear-type used for reported FIM sampling efforts, the gear-type categorical variables were associated with the SEAMAP database only.

## Results

### Analysis of Operating-Assessment Model Agreement

SEAMAP and CAGES year-pattern sampling methods produced unique fishery management index results per functional group, as detailed online [40]. The number of SEAMAP and CAGES FIM samples taken per year-pattern is shown in Table 2. Overall, the assessment model estimates agreed with known values of the operating model. Moreover, the operating model estimates of F_MSY_ agreed with historic, current and/or suggested fishing mortality rates, including Maximum Fishing Mortality Thresholds (MFMT) and other management reference values for GOM and the South Atlantic as calculated from previously published single-species stock assessments [40]. This suggests that the assessment model is an adequate tool for stock assessment in our simplified virtual ecosystem and offers a suitable portrayal of stock distributions and productivity. Potential exceptions included F_MSY_ values for the aggregated functional groups of Mullet, Red Snapper and Sea Trout.

**Table 2:** Number of Ecospace cells populated per habitat for each habitat in the SEAMAP and CAGES FIM programs.

	SEAMAP						Cages	
Yearpattern	<10 m	11–50 m	51–100 m	101–200 m	201–1000 m	Reef	<10 m	11–50 m
**2000**	81	667	191	21	0	0	370	2
**2001**	63	596	162	22	4	0	403	2
**2002**	75	668	194	27	2	2	408	1
**2003**	79	624	143	22	1	0	410	2
**2004**	81	641	170	14	0	0	406	2
**2005**	84	642	150	22	3	3	448	2
**2006**	93	607	208	56	10	1	60	1
**2007**	85	619	140	11	1	1	60	1
**2008**	73	785	299	22	1	0		
**2009**	101	1161	309	15	1	0		
**2010**	96	902	242	20	1	0		
**2011**	47	412	99	2	0	0		
**2012**	43	97	9	1	0	0		

### Most Effective Habitats for FIM Sampling

Comparison of management index estimates from the operating and assessment models revealed that the <10 m habitat most successfully determined MSY (see Fig 1 as <10 m area is extensive; RDA results with square root transformation to down-weight high abundance groups: R^2^ = 0.85). Although this R^2^ value was high, it primarily represented the habitat affinity for the functional groups of Jacks (R^2^ = 0.85), Menhaden (R^2^ = 0.25), Pin Fish (R^2^ = 0.77), Scaled Sardine (R^2^ = 0.94), and Stone Crab (R^2^ = 0.14). Similarly, square root transformed B_MSY_ was most accurately determined from Reef habitats (R^2^ = 0.36), although the R^2^ value primarily represents the functional group of Scaled Sardine. Inshore areas (<10 m) and reef habitats therefore emerge as the most important areas sampled, providing highly representative information on stock status and effectively supporting stock assessment models. Year-patterns that sampled in those regions tended to provide highly representative stock information for the above species. Overall F_MSY_ was not successfully determined by any single habitat; however, F_MSY_ was successfully determined for several individual functional groups. The most relevant information came from the following habitats: <10 m for Jacks (R^2^ = 0.88) and Pin Fish (R^2^ = 0.77), and <10 m and Reef for Scaled Sardine (R^2^ = 0.83). As the majority of CAGES data (~99%) came from the <10 m habitat, individual fishery indices with regards to habitat were not examined using RDA.

### Most Effective Gear-Types

Comparison of management index estimates from the operating and assessment models revealed that MSY was not successfully determined by the sampling effort for any particular gear-type. Instead MSY was most successfully determined for individual functional groups with the sampling efforts of three gear-types: Shrimp Trawls for Atlantic Croaker (RDA results: R^2^ = 0.31), Pin Fish (R^2^ = 0.30), Red Snapper (R^2^ = 0.30), Sea Trout (R^2^ = 0.20), and Stone Crab (R^2^ = 0.14), a combination of Shrimp Trawls and Experimental Shrimp Trawls for Scaled Sardine (R^2^ = 0.71), Experimental Shrimp Trawls for Mullet (R^2^ = 0.34), and Fish Trawls for Catfish (R^2^ = 0.50), Grouper (R^2^ = 0.64), and Ladyfish (R^2^ = 1.00). Overall F_MSY_ was not successfully determined by sampling effort of any one gear-type; however, F_MSY_ was successfully determined when fishing with gear-types unique to individual functional groups. The most relevant information came from the following gear-types: Shrimp Trawls for Atlantic Croaker (R^2^ = 0.34), Bay Anchovy (R^2^ = 0.34), Blue Crab (R^2^ = 0.17), Pigfish (R^2^ = 0.26), Sea Trout (R^2^ = 0.26), and Silver Perch (R^2^ = 0.26), a combination of Experimental Shrimp Trawls and Fish Trawls for Stone Crab (R^2^ = 0.55), and Fish Trawls for Catfish (R^2^ = 0.45), Grouper (R^2^ = 0.62), Ladyfish (R^2^ = 0.50), and Red Snapper (R^2^ = 0.17).

### FIM Sampling Accuracy Compared to Group Exploitation Status

**Fig 2:**
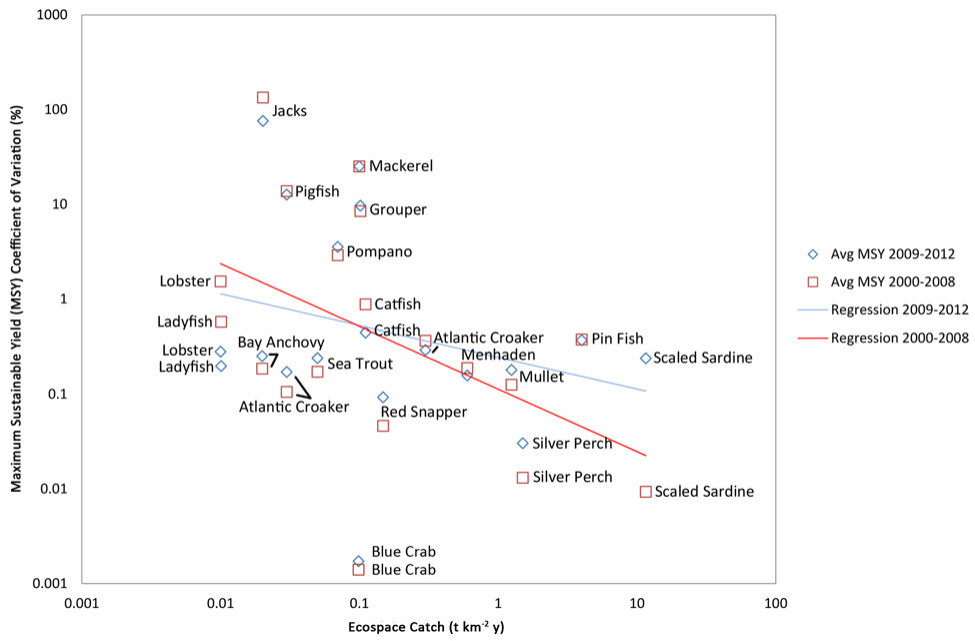
Accuracy of assessment model versus total catch by functional group. This figure illustrates the discrepancy (coefficient of variation) of Maximum Sustainable Yield (MSY) estimated between the (assessment) Pella-Tomlinson model and (operating) Ecospace model based on 2000 to 2008 data (red squares and regression line) and 2009 to 2012 data (blue diamonds and regression line). Catch quantities are as indicated by the Ecospace model from Walters et al. (2008). In general, exploited functional groups towards the top of the figure should be assessed by FIM programs with greater rigor and accuracy than other functional groups, as these functional groups have the highest coefficients of variance. Both regression line slopes were significantly different from zero (t-test: 2000 to 2008 P-value = 0.02, 2009 to 2012 P-value = 0.05), and from one another (t-test: P = 0.01).

**Fig 3:**
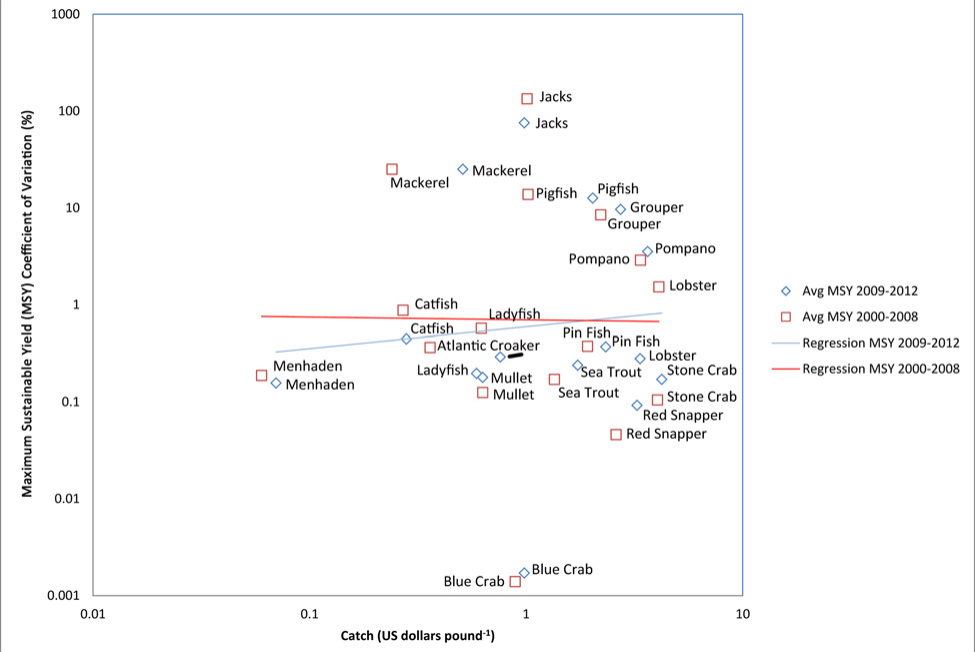
Accuracy of assessment model versus total catch value by functional group. This figure illustrates the discrepancy (coefficient of variation) of Maximum Sustainable Yield (MSY) estimated between the (assessment) Pella-Tomlinson model and (operating) Ecospace model based on 2000 to 2008 data (red squares and regression line) and 2009 to 2012 data (blue diamonds and regression line). Catch values ($ pound^-1^) per species are detailed by the National Oceanic and Atmospheric Administration (NOAA) online at: http://www.st.nmfs.noaa.gov/pls/webpls/FT_HELP.SPECIES. In general, valuable functional groups (towards the right of the figure) should be assessed by FIM programs with greater rigor and accuracy than other functional groups, particularly Grouper, Jacks, Mackerel, Pigfish, and Pompano. Regression lines are not significantly different from zero or one another.

**Fig 4:**
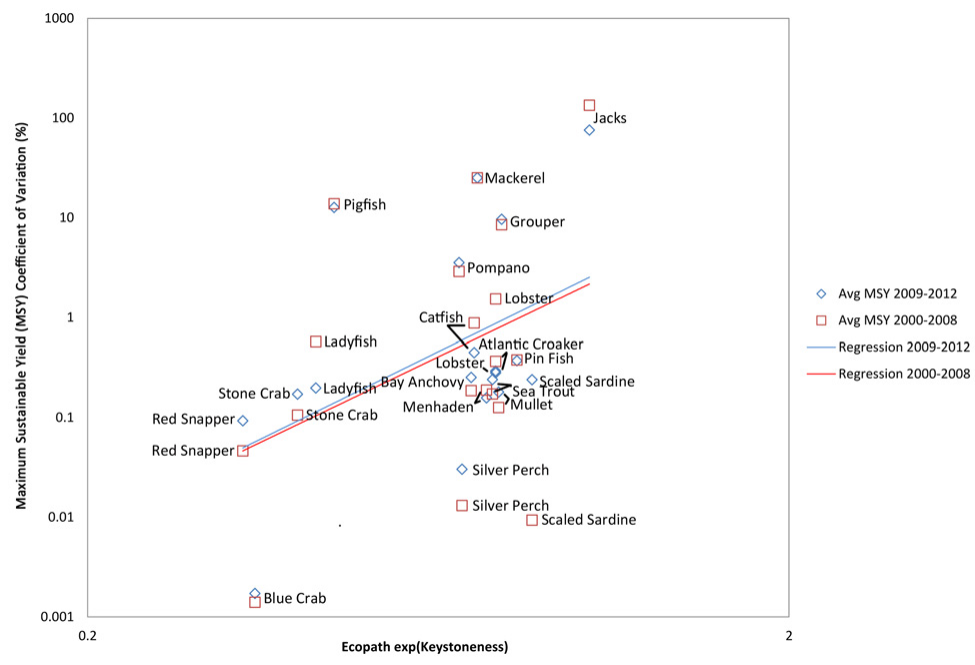
Accuracy of assessment model versus exponential Keystoneness by functional group. This figure illustrates the discrepancy (coefficient of variation) of Maximum Sustainable Yield (MSY) estimated between the (assessment) Pella-Tomlinson model and (operating) Ecospace model based on 2000 to 2008 data (red squares and regression line) and 2009 to 2012 data (blue diamonds and regression line). Keystoneness values were calculated by the Ecospace model. In general, ecologically important functional groups (towards the right of the figure) should be assessed by FIM programs with greater rigor and accuracy than other functional groups, providing a negative slope overall. That is not the case. Regression line slopes were significantly different from zero (t-test: 2000 to 2008 P-value = 0.05, 2009 to 2012 P-value = 0.01; values log-transformed), but not one another.

Fig 2 reveals the discrepancy (coefficient of variation) of MSY estimated between the assessment and operating models based on 2000 to 2008 data and 2009 to 2012 data versus Ecospace catch values (tonnes km^-2^ year^-1^), which includes discards/bycatch. The years from 2009 to 2012 illustrated differences in FIM data for reasons discussed below, and therefore Figs 2, 3 and 4 were divided into results of the years before and after 2009. One might expect a priori that exploited species with high coefficients of variance towards the top in Fig 2 should be assessed by FIM programs with greater rigor than exploited species with a low coefficient of variance towards the bottom of the figure. Therefore, we expect to see a negative slope where accuracy of the stock assessment improves for species with large total catch. In general, that is the case, specifically for the functional groups of Mullet, Pin Fish, Scaled Sardine, and Silver Perch. Exploited functional groups with the least amount of accuracy are Grouper, Jacks, Mackerel, Pigfish and Pompano. With some important exceptions, the sampling efforts from 2009 to 2012 were less effective in producing accurate management index estimates than years’ previous, as demonstrated in the regression line’s more positive slope when compared to the 2000 to 2008 regression line. Both regression line slopes were significantly different from zero (t-test; 2000 to 2008 P-value = 0.02, 2009 to 2012 P-value = 0.05), and from one another (t-test; P = 0.01). These exceptions indicate that programmatic or technical changes to FIM since 2009 in the SEAMAP program have reduced accuracy for assessing high biomass fish populations in the GOM.

### FIM Sampling Accuracy versus Value and Ecological Importance

Using the discrepancies of MSY estimated between the assessment and operating models of the 2000 to 2008 and 2009 to 2012 data, Figs 3 and 4 revealed that the FIM sampling provides the least accurate information for some of the most economically valuable and ecologically important functional groups. These functional groups include Grouper, Jacks, Mackerel, Pigfish and Pompano. These conclusions accompany regression line slopes in Fig 3 that are not significantly different from zero or one another, when comparing 2000 to 2008 with 2009 to 2012, and indicate that FIM sampling produced equally poor assessments of MSY for many economically valuable functional groups (e.g., Red Snapper, Sea Trout and Stone Crab), regardless of catch value. The ecologically important functional groups in Fig 4 (e.g., Mullet, Scaled Sardine, Silver Perch) and regression line slopes indicates that FIM sampling was equally poor for the time periods before and after 2009, and least accurate ecologically important functional groups. Fig 4 regression line slopes were significantly different from zero (t-test; 2000 to 2008 P-value = 0.05, 2009 to 2012 P-value = 0.01; values log-transformed), but not one another. The effort of FIM programs by species is therefore not allocated proportionally to the value of stocks from an economic perspective or the importance of stocks from an ecological perspective.

### Statistical Analysis of Raw FIM Catch Data

**Fig 5:**
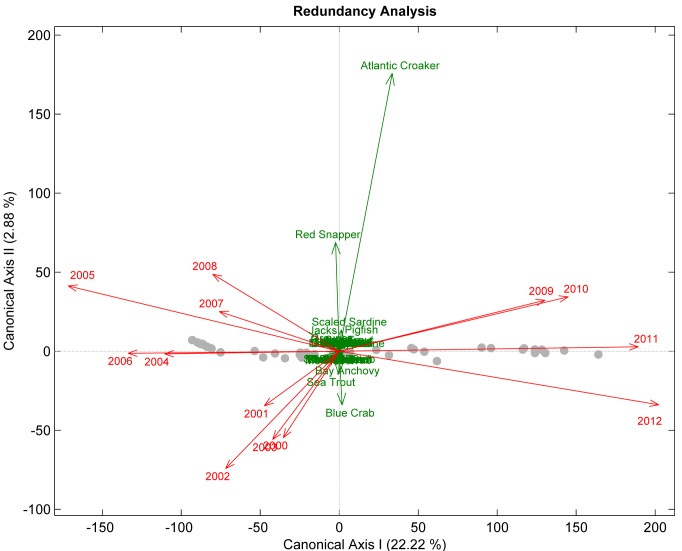
Redundancy Analysis Plot of Year and CPUE per Functional Group. Redundancy analysis shows the functional groups (green text and vectors) most associated with the programmatic changes in the SEAMAP program according to the year-pattern (red text and vectors). The year-pattern refers to the geographical locations described as points in the operating model. Approximately 25% of the variability in functional group abundances is explained by the sampling year-pattern in two canonical axes. In general post-2009 changes have benefited only a few species, in particular Atlantic Croaker.

The most representative SEAMAP sampling years between 2000 to 2012 that explained the most variability in CPUE for a few select species, and presented in order of variable selection and cumulative R^2^ values, are: 2012 (R^2^ = 0.05), 2010 (R^2^ = 0.11), 2009 (R^2^ = 0.19), and 2011 (R^2^ = 0.33). This is due to reduced discrepancies between the operating and assessment model for primarily the functional groups of Atlantic Croaker, Blue Crab, Catfish, Jacks, Menhaden, and Pigfish (Fig 5). Reduced discrepancies indicate that sampling strategies in the most recent years were more effective at providing representative stock information for the few functional groups listed above; however, sampling strategies might be less efficient for other functional groups (e.g. Red Snapper, Scaled Sardine or Silver Perch; Fig 2), and/or variability in CPUE could not be explained by sampling strategies used from 2000 to 2008 compared to those from 2009 to 2012. CAGES results by Gulf state showed that the most valuable sampling year(s) in Alabama were 2006 (R^2^ = 0.03) and 2007 (R^2^ = 0.07), Florida 2003 (R^2^ = 0.04), and Louisiana 2007 (R^2^ = 0.15) and 2001 (R^2^ = 0.19). Therefore, there has not been a noticeable increase or decrease in the quality of information emerging from CAGES; sampling years that provided the best results were scattered among the history of the CAGES program. Similarly, RDA tests for Mississippi and Texas revealed no differences in CAGES sampling variability from year-to-year.

**Fig 6:**
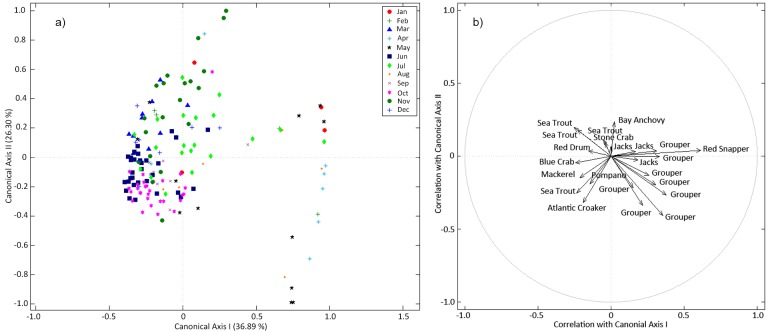
Canonical analysis of principal coordinates (CAP) point (a) and vector (b) plots for SEAMAP abundance data organized by sampling months per year. Axes in a) are labelled according to the overall classification accuracy by sampling month (78%), whereas axes in b) depict the correlations to sampling month(s) by vector length along the first two canonical axes depicted in the CAP point-plot. Vector length is proportional to a month’s contribution in separating functional groups by abundance, where longer vector lengths indicate importance. In general this figure illustrates a predictable assemblage of species based on monthly captures, and indicates the similarity of different months in terms of the assemblage caught and therefore information content.

The relative abundances of SEAMAP species comprising the 35 evaluated functional groups were found to be significantly different between sampling months (np-Manova; F = 4.10, P = 0.001, N = 162), suggesting that certain months may provide more comprehensive information by species than other months. CAP tests revealed that sampling months had an overall 78% classification accuracy rate when classifying data (Fig 6), which was significantly better than the 12% success rate predicted by PCC (P = 0.001). Therefore, sampling month has a strong effect in the quality of information produced by the SEAMAP program. When vector plots were examined, information overlap appeared most evident for Atlantic Croaker, Blue Crab, Pompano, Mackerel, and Sea Trout, and less evident for Bay Anchovy, Jacks, Red Drum, and Stone Crab. Species of Grouper, Red Snapper and two species of Jacks clearly showed that their abundance data was limited to specific sampling months, and no overlap with other sampling months was observed. The timing of sampling for these species is therefore inflexible and should be directed towards the most effective months.

RDA and IV results of relative abundances according to sampling month were then detailed by species and functional group in Table 3, although not all species in the functional groups were found to have clearly important explanatory months and/or a month affinity values. RDA tests demonstrated that the majority of the species that had important explanatory variables were caught most often in a single sampling month, or over a seasonal sampling period (two or more consecutive sampling months). In a few cases the species’ R^2^ value indicated it was caught biannually. In general, R^2^ values from RDA tests on single species ranged from 0.01 to 0.50, and IV ranged from 0.0% to 72.5%. It should be noted that the IV percentages reported in Tables 3, 4 and 5 can be read additively if two or more values are reported for a single species. An example of this was observed with the Blue Crab, *Callinectes sapidus*, whose IV percentages for July and June were 30.0 and 26.0%, respectively, which indicated that 56% of the time this species was caught during these months.

**Table 3:** Analysis of species-specific historic SEAMAP catch data using Redundancy Analysis with Akaike information criterion results for month and Indicator Values (IV) (numbers).

Functional Group	Species	Jan	Feb	Mar	Apr	May	Jun	Jul	Aug	Sep	Oct	Nov	Dec
Atlantic Croaker	*Micropogonias undulatus*										*		
Bay Anchovy	*Anchoa mitchilli*							*					
Blue Crab	*Callinectes spp*.										*		
	*Callinectes sapidus*						*26*.*0*	*30*.*0*					
	*Callinectes similis*						*17*.*6*	*17*.*2*			*		
Catfish	*Bagre marinus*											*17*.*9*	
Grouper	*Epinephelus adscensionis*				20.0								
	*Epinephelus drummondhayi*				14.4	*							
	*Epinephelus flavolimbatus*				35.1	*							
	*Epinephelus morio*					13.3							
	*Epinephelus nigritus*		23.4										
	*Epinephelus niveatus*				19.0	*							
	*Mycteroperca interstitialis*					*							
	*Mycteroperca microlepis*					15.9							
	*Mycteroperca phenax*		15.6		*	*							
Jacks	*Caranx crysos*					*					*		
	*Caranx hippos*							*		47.4			
	*Caranx latus*							*					
	*Seriola dumerili*							*					
	*Seriola rivoliana*				*								
	*Seriola zonata*				*								
Ladyfish	*Elops saurus*								*	*18*.*4*	*		
Lobster	*Munida spp*.								*				
	*Munida fliniti*										*		
	*Munida forceps*			*									
	*Munida irrasa*									**			
	*Munidopsis spp*.			*							*		
	*Munidopsis robusta*			18.7									
	*Nephropsis aculeata*		*13*.*7*										
	*Polycheles spp*.			16.1									
	*Scyllarides nodifer*									*			
	*Scyllarus spp*.						*						
	*Scyllarus chacei*						*12*.*3*						
	*Scyllarus depressus*										*14*.*1*		
Mackerel	*Scomber japonicus*			*30*.*2*									
	*Scomberomorus cavalla*						*14*.*7*		**		*14*.*1*		
	*Scomberomorus maculatus*						*18*.*1*	*			*20*.*6*		
Menhaden	*Brevoortia patronus*									*29*.*1*			
Mullet	*Mugil cephalus*						*						
	*Mugil curema*						*						
Pigfish	*Orthopristis chrysopterus*							*			*		
Pin Fish	*Diplodus holbrooki*										*		
Pompano	*Rachycentron canadum*										**		
	*Trachinotus carolinus*										30.0		
Red Drum	*Sciaenops ocellata*						*				*13*.*0*		
Red Snapper	*Lutjanus campechanus*	38.7			*18*.*3*	*							
Functional Group	Species	Jan	Feb	Mar	Apr	May	Jun	Jul	Aug	Sep	Oct	Nov	Dec
Scaled Sardine	*Harengula jaguana*					*							
Sea Trout	*Cynoscion spp*.										**72.5**		
	*Cynoscion arenarius*												**18.9**
	*Cynoscion nebulosus*		14.1	*									
	*Cynoscion nothus*	*			*	*		*					
Stone Crab	*Menippe adina*					*							
	*Menippe mercenaria*							*16*.*0*					

The italicized IV number or

* denotes R^2^ values for the month between 0.01 and 0.09, the underlined IV number or

** between 0.10 and 0.29, and the bold IV number or

*** >0.30. Thus, a bold number or

*** indicates that the month consistently yields information for the species being surveyed. The IV represents the faction or percent of time the species was caught in the sampling month, indicating the irreplaceability of that month in sampling the species. Only IV values with significant P-values (<0.05) were reported.

**Table 4:** Analysis of species-specific historic SEAMAP catch data using Redundancy Analysis with Akaike information criterion results for gear-type (color) and Indicator Values (IV) (numbers).

Functional Group	Species	BB	BL	ES	FT	HO	OB	SM	ST	TR	TV	VC
Atlantic Croaker	*Micropogonias undulatus*			57.8					***			
Bay Anchovy	*Anchoa mitchilli*								**78.2**			
Blue Crab	*Callinectes spp*.					**99.0**						
	*Callinectes sapidus*								**68.6**			
	*Callinectes similis*				*26*.*6*							
Catfish	*Bagre marinus*								***			
Grouper	*Epinephelus drummondhayi*				**							
	*Epinephelus flavolimbatus*		***				*35*.*3*					
	*Epinephelus morio*				**							
	*Epinephelus niveatus*		***									
	*Mycteroperca interstitialis*										**	
	*Mycteroperca microlepis*										**	
	*Mycteroperca phenax*										**	
Jacks	*Caranx spp*.							**99.7**				
	*Caranx crysos*			**97.3**								
	*Caranx hippos*								**			
	*Seriola dumerili*						**70.8**			**		
	*Seriola fasciata*										**	
	*Seriola rivoliana*									**		
Lobster	*Munida spp*.				**							
	*Munida pusilla*								**			
	*Nephropsis aculeata*	**94.6**										
	*Polycheles spp*.	87.6										
	*Scyllaridae spp*.					**99.8**						
	*Scyllarides nodifer*								*			
	*Scyllarus chacei*								*			
Mackerel	*Scomber japonicus*				***							
	*Scomberomorus maculatus*			56.*4*	**							
Menhaden	*Brevoortia patronus*				*				**			
Pigfish	*Orthopristis chrysopterus*								**			
Pin Fish	*Diplodus holbrooki*								*			
Pompano	*Trachinotus carolinus*				*23*.*2*				*			
Red Drum	*Sciaenops ocellata*								**			
Red Snapper	*Lutjanus campechanus*										**77.7**	
Scaled Sardine	*Harengula jaguana*			**75.8**					**			
Sea Trout	*Cynoscion spp*.			**					**			
	*Cynoscion arenarius*								**42.6**			
	*Cynoscion nothus*								46.6			

The italicized IV number or

* denotes R^2^ values for the gear-type between 0.01 and 0.09, the underlined IV number or

** between 0.10 and 0.29, and the bold IV number or

*** >0.30. Thus, a bold number or

*** indicates that the gear-type consistently yields information for the species being surveyed. The IV represents the faction of time the species was caught with the sampling gear-type, indicating the irreplaceability of that gear-type in sampling the species. Only IV values with significant P-values (<0.05) were reported. Gear-type abbreviations are: Bib Trawl (BB), Bottom Longline (BL), Experimental Shrimp Trawl (ES), Fish Trawl (FT), Hand Line (HL), High Opening Bottom Trawl (HO), Off-bottom Longline (OB), Standard Mongoose Trawl (SM), Shrimp or SEAMAP Trawl (ST), Fish Trap (TR), Video Trap (TV), and Video Camera (VC).

**Table 5:** Analysis of species-specific historic CAGES catch data using Redundancy Analysis results with Akaike information criterion results for month (color) and Indicator Values (IV) (numbers).

Functional Group	Species	Gulf State	Jan	Feb	Mar	Apr	May	Jun	Jul	Aug	Sep	Oct	Nov	Dec
Atlantic Croaker	*Micropogonias undulatus*	Texas		*	*	18.3	19.6	14.4	*	*	*			
		Louisiana	16.6	18.9	22.1	19.2	*							
		Alabama		*17*.*3*	*21*.*7*	*14*.*9*	*							
		Florida			*									
Bay Anchovy	*Anchoa mitchilli*	Texas	19.0	*13*.*9*									*	*
		Louisiana				*						*10*.*3*		
		Alabama								*15*.*5*	*12*.*5*			
		Florida								*				
Blue Crab	*Callinectes sapidus*	Texas	*	*	*15*.*0*	*15*.*8*	*14*.*4*	*11*.*1*	*					
		Louisiana	*14*.*3*	*15*.*1*	*12*.*2*	*	*	*						*
		Alabama			*17*.*3*	*15*.*9*	*11*.*9*	*						
		Florida			13.5	*	*							
Blue Crab	*Callinectes similis*	Texas												
		Louisiana					*							
		Alabama						*						
Catfish	*Ariopsis felis*	Texas	**	**					*13*.*7*	*12*.*7*	*12*.*3*		*	**
		Louisiana							*17*.*7*	**30.7**	*17*.*6*	*		
		Alabama									*20*.*3*	*		
Catfish	*Bagre marinus*	Alabama							*49*.*2*	12.3	*			
Catfish	*Ictalurus furcatus*	Alabama		*										
Catfish	*Ictalurus punctatus*	Alabama		*										
Jacks	*Caranx crysos*	Louisiana					*							
		Alabama		*								*		
Jacks	*Caranx hippos*	Louisiana						37.9	34.1	10.8				
		Alabama						*27*.*0*	**					
Jacks	*Hemicaranx amblyrhynchus*	Louisiana							*12*.*2*	*15*.*8*			*	
		Alabama								*14*.*0*	*	*		
Ladyfish	*Elops saurus*	Louisiana			*	*		*	*24*.*2*	*11*.*2*				
		Alabama		*19*.*4*		*	*25*.*5*							
Mackerel	*Scomberomorus cavalla*	Louisiana							*					
		Alabama								*				
Mackerel	*Scomberomorus maculatus*	Texas						*	*11*.*3*	*	*11*.*8*	*		
		Louisiana					*	37.1	30.0	*11*.*8*	*			
		Alabama						*						
		Florida						*						
Menhaden	*Brevoortia patronus*	Texas	*15*.*9*	*16*.*3*										
		Louisiana	*17*.*8*	23.0	*23*.*5*	*16*.*5*	*							
		Alabama			*20*.*4*									
		Florida		*	*									
Menhaden	*Brevoortia smithi*	Alabama	*											
Mullet	*Mugil cephalus*	Texas	32.2	24.3										*20*.*8*
		Louisiana	28.3	*16*.*4*	*								*	*18*.*2*
		Alabama			*	*								
		Florida	**											
Mullet	*Mugil curema*	Louisiana							*14*.*2*	29.9	*			
		Alabama	*											*
Functional Group	Species	Gulf State	Jan	Feb	Mar	Apr	May	Jun	Jul	Aug	Sep	Oct	Nov	Dec
Pigfish	*Orthopristis chrysoptera*	Louisiana						*						
		Alabama				*								
Pin Fish	*Lagodon rhomboides*	Texas											*	*12*.*1*
		Louisiana					*	20.3	22.4	*17*.*3*	*	*		
		Alabama			*22*.*0*	*								
Pompano	*Trachinotus carolinus*	Louisiana				*								
		Alabama						*	*					
Red Drum	*Sciaenops ocellatus*	Texas	*16*.*6*	*										*13*.*4*
		Louisiana				*								
		Florida											*	*
Red Snapper	*Lutjanus campechanus*	Louisiana								*				
		Alabama								*23*.*6*	*16*.*9*	*		
Scaled Sardine	*Harengula jaguana*	Louisiana							*15*.*0*	30.2	24.7			
		Alabama				*								
Sea Trout	*Cynoscion arenarius*	Texas					*	*15*.*4*	15.0	14.9	*12*.*1*	*	*	*13*.*6*
		Louisiana				*	18.8	23.1	18.2	*	*	*		
		Alabama				*27*.*1*	*17*.*2*	*						
		Florida					*	*	*	*	*	*		
Sea Trout	*Cynoscion nebulosus*	Texas	*14*.*0*	*										*11*.*0*
		Louisiana	**	*	*								*	**
		Alabama								*				
Sea Trout	*Cynoscion nothus*	Louisiana											*	
		Alabama									*18*.*9*	*19*.*9*		
Silver Perch	*Bairdiella chrysoura*	Texas		*	*	*								
		Louisiana						*16*.*9*	*20*.*0*	*16*.*1*				
		Alabama							*		*			
		Florida			*									
Stone Crab	*Menippe adina*	Louisiana											*	
		Alabama			*									
Stone Crab	*Menippe mercenaria*	Louisiana			*									
		Alabama												*

The italicized IV number or

* denotes R^2^ values for the month between 0.01 and 0.09, the underlined IV number or

** between 0.10 and 0.29, and the bold IV number or

*** >0.30. Thus, a bold number or

***indicates that the month consistently yields information for the species being surveyed. The IV represents the fraction of time the species was caught in the sampling month, indicating the irreplaceability of that month in sampling the species. Only IV values with significant P-values (<0.05) were reported. Table summarizes species into EwE functional groups for comparison.

**Fig 7:**
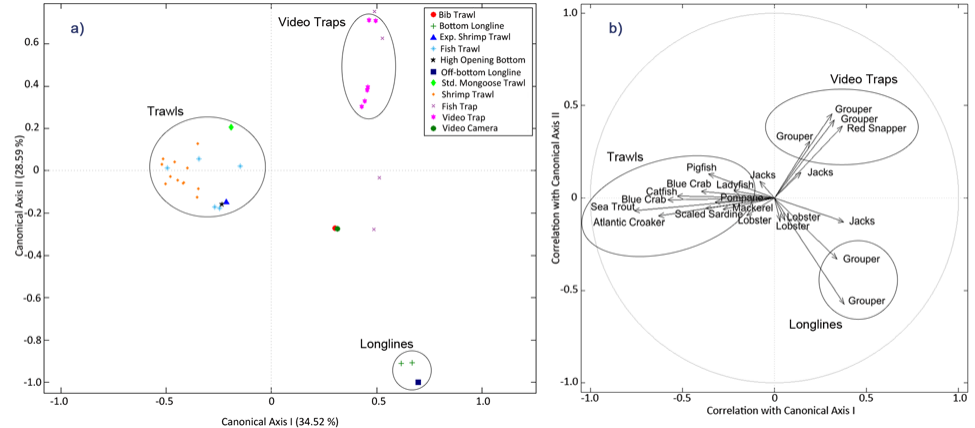
Canonical analysis of principal coordinates (CAP) point (a) and vector (b) plots for SEAMAP abundance data organized by sampling gear-types used each month. Axes in a) are labelled according to the overall classification accuracy by sampling gear-type (89%), whereas axes in whereas axes in b) depict the correlations to gear-types by vector length along the first two canonical axes depicted in the CAP point-plot. Vector length is proportional to gear-type(s) contributions in separating functional groups by abundance, where longer vector lengths indicate importance. In general this figure illustrates a predictable assemblage of species based on gear-type captures, and indicates the similarity of different gear-types in terms of the assemblage caught and therefore information content.

Relative abundances of the SEAMAP species comprising the 35 evaluated functional groups were also found to be significantly different when sampling with different gear-types (np-Manova; F = 3.94, P = 0.001, N = 34), and so the second null hypothesis was rejected: certain gear-types may provide more comprehensive information than other gear-types. CAP tests revealed that gear-type had an overall 89% classification accuracy rate when classifying data (Fig 7), which was significantly better than the 19% success rate predicted by PCC (P = 0.001). A gear-type CAP plot illustrated in Fig 7 indicated that functional groups were mostly captured using one to two gear-types.

RDA and IV results of relative abundance according to sampling gear-type were then detailed by species and functional groups in Table 4, although not all species were found to have particularly important explanatory gear-types and/or a gear-type affinity values. RDA tests revealed that all species had one or two most important gear-type(s) from which abundance data was determined. For instance, the Grouper (e.g. *Epinephelus flavolimbatus*), Red Snapper (*Lutjanus capechanus*), and Jacks (e.g. *Caranx crysos*) abundance data was most often determined from Longlines (BL and OB), Video Traps (TV), and Experimental Shrimp Trawl (ES), respectively. In general, R^2^ values from RDA tests on a single species ranged from 0.04 to 1.00, and IV ranged from 0.0% to 99.8%. The average of the significant IV percentages among species in Table 4 was 67.8%, which signified that many species had a high group affinity to one particular gear-type.

The CAGES database had similar species among the five Gulf States; however, no state database contained all of the species that were considered in SEAMAP database analyses, and the total number of species and identity also varied by Gulf state. Initial statistical analyses by Gulf State indicated that we could not reject the first null hypothesis for Mississippi: all months appear equally suited to provide abundance data. However, the first null hypothesis was disproved for Alabama (np-Manova; F = 9.76, P = 0.001 based on 34 species), Florida (np-Manova; F = 2.49, P = 0.001 based on 10 species), Louisiana (np-Manova; F = 22.85, P = 0.001 based on 49 species), and Texas (np-Manova; F = 19.58, P = 0.001 based on 12 species) as relative abundances of CAGES species were significantly different between sampling months.

CAP tests revealed that the majority of variability in species abundances, as sampled by individual Gulf states, was accurately classified by sampling months. In Alabama categorical sampling month had an overall 64% classification accuracy rate, Florida a 76% classification accuracy rate, Louisiana 91% a classification accuracy rate, and Texas a 93% classification accuracy rate. Each Gulf state also had a classification accuracy rate that was significantly better than the success rate predicted by PCC (P = 0.001). Gulf state results henceforth are presented according to geographical positions, west to east, starting from Texas and ending with Florida (Table 5). This allowed for the presentation of insights per species in terms of potential seasonal changes associated with individual Gulf States and ecosystem dynamics. As with SEAMAP data, a large majority of variability in species’ abundances could be explained by sampling month.

Not all species were found to have particularly important explanatory months and/or month affinity values. Similar to what was observed in SEAMAP results, the majority of the species caught by the CAGES program that had clear explanatory months were caught most often over a seasonal sampling period. The most informative seasonal sampling periods generally occurred earlier in seasonal cycles and/or included more sampling months. This was true in Texas and Louisiana, whereas there were fewer seasonal sampling periods and less information for Alabama and Florida. In general R^2^ values for the CAGES program ranged from 0.01 to 0.63, and IV ranged from 0.0% to 37.9%.

## Discussion

In our efforts to understand the total scientific benefit of the SEAMAP and CAGES databases we quantified the value of these 14 FIM programs using simulated FIM sampling from an operating model (Ecospace) and feeding the results to a separate stock assessment model (Pella-Tomlinson). The stock assessment model was then used to estimate fisheries management indices for 35 functional groups. The accuracy of the assessment model was compared to known index values from the operating model, obtained using a manual equilibrium analysis, and to previously published single-species stock assessments. In stock assessments certain year-patterns performed better than others for predicting the true management indices reflecting differences in how representative FIM data were for judging stock health based on particular areas/habitats/depths that were sampled in those years, and the relative sampling effort of various sampling gear-types. A redundancy analysis teased out what characteristics made each year’s sampling pattern particularly apt at providing the most relevant information to the stock assessment process; a method which can help guide reallocation of sampling effort between FIM programs, and times and areas sampled. With these efforts we conclude with insights into estimating fishery management indices based on trophodynamics considerations, demonstrating the ability of FIM programs to maximize scientific benefit.

Results from the operating and assessment models indicated that the majority of fishery indices for the 35 functional groups evaluated in the SEAMAP and CAGES programs could most correctly be determined by sampling near-shore habitats and reefs. However, year-pattern sampling efforts in both SEAMAP and CAGES clearly affected the estimated biomass values, and so refining future sampling efforts would likely lead to more accurate stock assessments of managed species. In general, lower biomass species should be assessed by FIM programs with greater rigor. Consequently we expected to have less accurate estimates of their biomass, and the estimates of MSY made by the assessment model should not have closely matched the true MSY values from Ecospace. Indeed, that was the case. We see a reliable trend in Fig 2, where estimates of MSY from the assessment model matched the true value of the operating model with greater precision for higher biomass groups than for lower biomass groups. This was particularly true for the high biomass groups of Atlantic Croaker, Menhaden, Mullet, and Silver Perch, and low biomass groups of Grouper, Jacks, Mackerel, Pigfish and Pompano. Although effort in the FIM programs seem to reflect, approximately, the total tonnage caught, the effort in FIM programs does not similarly reflect the relative value of fisheries (Fig 3), or the ecological importance of species (Fig 4). These graphs can help in the prioritization of species for additional monitoring. The best months and gear-types to use in this pursuit are explored in Tables 3, 4 and 5.

For example the high catch value of Grouper, Jacks, Lobster, Mackerel, Pigfish and Pompano (Fig 3), as well as their ecological importance in the GOM ecosystem (Fig 4) should further encourage expanded monitoring of these functional groups. Another important GOM functional group included Scaled Sardine, which revealed far less effective FIM-based management for these species (which are located on the bottom right of Figs 2 and 4) would improve allocation of FIM effort both in terms of stock value and the ecology of the system. Grouper, Jacks, Mackerel, Pigfish and Pompano are all piscivorous functional groups, and are recommended for increased assessment efforts. Not only do these functional groups support important commercial fisheries, they likely play an important trophodynamic role as predators of the forage base in the GOM, and so may directly influence bait fisheries. Thus, they may be more valuable than the forage base based on catch value or keystoneness, and may be good candidates for expanded monitoring, especially in near-shore habitats.

With several exceptions, the SEAMAP sampling efforts from 2009 to 2012 were less effective in producing accurate biomass than previous years’ estimates, providing less precise estimates of MSY from the assessment model, and providing the least accurate (or representative) information on species’ relative abundances. Exceptions are noted for Catfish, Ladyfish, and Lobster (Figs 2 to 4). This indicated that programmatic or technical changes to FIM since 2009 have not paid dividends in an improved ability to assess fish stocks in the GOM. Note that this result was seen in both the comparison of the assessment and operating models, and also in the statistical analysis of raw FIM catch data. In the former case, year-patterns before 2009 provided the most accurate simulated sampling data with which to drive the assessment model, and in the latter case FIM data after 2009 was less representative of the long-term average CPUE in the ecosystem. Interestingly, the SEAMAP Shrimp Trawl sampling protocol changed in 2009 from sampling across depth stratum to a 30-minute fixed tow time, which also included sampling day and night, although diel movement was not considered here [63]. As the SEAMAP Shrimp Trawl provided the greatest amount of information regarding species’ relative abundances in the present study, and was used ~99% of the time for sampling functional group species between 2000 and 2012, it was obviously an integral gear-type when compared to the other 12 SEAMAP gear-types, but other gear-types should be used more frequently.

When the raw historic catch for SEAMAP’s monthly sampling strategies were analyzed statistically, we could further suggest which months could yield the greatest improvement in the accuracy of stock assessments. Suggestions were made based on observed species’ abundances over two or more months. These increased abundances were thought to be attributable to ecological factors, including ontogenic migrations. For example, according to the SEAMAP database Ladyfish (*Elops saurus*) was observed to have higher abundances during the warmer water months of August, September and October. When temperatures begin to decline in the GOM ecosystem, Ladyfish have been reported to migrate into deeper waters during colder seasonal periods [64], consequently becoming less abundant in other sampling months. In the present study, Ladyfish was an indicator species for the month of September based on IV (Table 3) in SEAMAP. For ladyfish, and other species (e.g. Red Drum and Red Snapper) the RDA and IV test findings reported in Table 3 were also supported by the CAP point and vectors plots in Fig 6. The significant groupings of species’ abundance according to sampling months in Fig 6 also provided evidence of informational overlap in the SEAMAP database and indicated several important sampling months for functional group species. For instance, informational overlap was observed between the months of June, September and October, signifying that for those months the majority of the species collected during samplings had similar abundances. Other seasonal observations could be categorized as biannual abundance increases, such as the spawning aggregations of the Red Drum (*Sciaenops ocellata*) in summer (June) and fall (October), which would increase abundances in localized areas during these two sampling months [65–68]. Red Drum was also selectively caught in October based on IV (Table 3). Although species of Grouper, Mackerel, Pompano, Sea Trout, as well as the Red Snapper and Ladyfish all had higher variability in relative abundance explained by the sampling month(s), other species showed less evident associations. Considering species-specific strategies for sampling monthly efforts is likely to enhance the SEAMAP program.

In addition to sampling month, the SEAMAP sampling gear-type(s) were even more successful in explaining the observed variability in species’ abundances. It became evident that increasing the use of certain sampling gear-types could also greatly enhance relative abundance reporting for several species. Examples included increasing the use of Video Traps to sample Grouper, Lobster, and Red Snapper, or increasing the use of Fish Traps to sample the Blue Crab, Lobster, and Mackerel. Examining catch at the species level clearly demonstrated that Grouper species were successfully captured most often with Longlines and Video Traps, while Atlantic Croaker was successfully captured most often with Shrimp Trawls or sometimes Experimental Shrimp Trawls. It was noted that the Shrimp Trawl gear-type was the most widely used among all gear-types between 2000 and 2012, used more than 99% of the time to capture all functional group species, and therefore provided abundance information for the greatest number of species. While Shrimp Trawls provided valuable information, Fig 7 indicated that several other gear-types were better suited for capturing important fishery managed species (i.e. Grouper and Red Snapper using Video Traps (TV)). The General Plankton (PN) and Hand Line (HL) gear-types in the SEAMAP database never captured any species in the functional groups in this study. Increasing the use of video monitoring may also allow for better accuracy in assessing near-shore and reef habitats, and reduce some relative abundance assignment confusion with Bib Trawls for the Lobster functional group, as well as increase access to habitats inaccessible to Shrimp Trawls.

Considering statistical results from SEAMAP database analyses, and monthly and gear-type areas of sampling overlap, Tables 3 and 4 together provide suggested combinations of ideal sampling months and gear-types for capturing functional group species in future sampling efforts. These month and gear-type combinations would be expected to increase the likelihood of collecting samples that more accurately reflect GOM stocks. Overall, SEAMAP sampling months from April to July, as well as the month of October, produced the most information for the majority of functional groups species. The months of February, March, August and September produced a moderate amount of information, and November through January produced the least. However, a few species of Crab, Grouper and Sea Trout exhibited significance with one of the less informative sampling months. For example, this was true for *Epinephelus nigritus* in February and *Cynoscion arenarius* in December (Table 3; Fig 6). This indicated that, although those particular sampling months were less important for many other species, they were most informative for determining these species’ relative abundances. Shrimp Trawl was the most important gear-type for sampling all functional groups, as it consistently provided the most information regarding species abundances. However is was noted that from 2000 to 2012 shrimp trawl rarely captured species in the functional groups of Ladyfish, Mullet, Pompano, Red Drum and Stone Crab.

The CAGES database revealed various sampling advantages and challenges encountered by each of the Gulf States. We analyzed this program state-by-state rather than program-wide because of variability in estuary productivity, estuary size, trawling gears used, and towing speeds. State-by-state differences were found between sampling years, as gradients in seasonal abundances were observed in GOM estuaries according to sampling months and according to species reported in each Gulf State database (Table 5). It was observed that the CAGES database, compared to the SEAMAP database, revealed clearer seasonal migrations, movements, and therefore changes in abundance according to ecological dynamics unique to each Gulf State and estuary. A likely example is the Blue Crab, *Callinectes sapidus*, which follows an inshore/offshore migration pattern (e.g. [69]) from estuaries to offshore waters [70,71], traveling from tens to hundreds of kilometers [72,73]. Supporting ecological dynamics unique to each Gulf State, *Callinectes sapidus* migration patterns vary by region [72–74], with evidence of significant genetic differences between the eastern (Texas and Louisiana) and the western (Alabama and Florida) GOM [75], and redistributions of larvae due to the GOM loop current [76].

Recommended CAGES program sampling months for functional groups and by Gulf State in Table 5 corresponded to many inshore/offshore migrations, which describe population movements to and from the estuaries. For instance, Sea Trout (*Cynoscion arenarius*) were most often in Texas from June to September, in Louisiana from May to July, and in Alabama from April to May. Some functional groups yielded similar insights, while others had less conclusive month associations as observed in the CAP axes and vectors plot. Examples include the: Atlantic Croaker, *Micropogonias undulates*, which are more abundant in northern waters during warmer months and migrate from estuaries into deeper waters for fall and winter spawning (e.g. [77,78]); Bay Anchovy, *Anchoa mitchilli*, which migrate out of estuaries and into lower latitudes during winter months [79]; Catfish, *Ariopsis felis*, which migrate to deeper, stable temperature waters in winter months, but in some nearshore waters remain year-round [80]; Mackerel, *Scombermorus maculatus*, which migrates thousands of kilometers seasonally along coastlines between Texas and Florida [81,82]; Menhaden, *Brevoortia patronus*, which migrates from estuaries to offshore waters from summer to winter [83]; Sea Trout, *Cynoscion arenarius*, which leaves estuaries in the fall and overwinter in the GOM [84]; and Stone Crab, *Menippe mercenaria* which are less abundant in nearshore waters during warmer months (e.g. [85]. These and other species have monthly sampling recommendations based on statistical analyses that align with species-specific ontogeny.

## Conclusion

Even using the best sampling methods there is inherent observational error associated with each FIM program. Observational error can develop from changes in locations or equipment from year-to-year, the omission of important age classes of a species (e.g. juveniles are not caught in larger mesh nets), or include problems associated with habitats that are difficult to sample (e.g. shrimp trawls cannot sample coral reefs). For these reasons it is compelling to use a whole-ecosystem analysis that can represent that data gathering capabilities of a suite of sampling gear-types simultaneously to look for gaps in information delivered to stock assessment and redundancies in the habitats, species and age classes sampled. Moreover, a dynamic whole-ecosystem analysis including spatial trophodynamics of multiple species and age classes, like the one performed here, also captures important predator-prey interactions and other indirect effects, and so accounts for some of the process uncertainty that lies between data gathering and the use of that data in stock assessments. As FIM programs are partially funded through taxpayer dollars, and fisheries receive subsidies from taxpayer dollars, dynamic ecological analysis of FIM sampling strategies has the potential to direct future FIM sampling efforts and provide more accurate ecological information from which to guide fishery regulations.

SEAMAP and CAGES are two distinct programs which collect details concerning many similar target species, but share only a few similarities in sampling equipment and sites. SEAMAP has the challenge of sampling near-shore waters as well as deeper waters off of the Gulf coast, while GAGES concentrates on sampling GOM estuaries from Texas to Florida. With dissimilarities in sampling extents it is likely that both programs will continue to have dissimilar sampling reports of the same target species. Likewise, in the present study we found that the SEAMAP and CAGES FIM programs had unique recommendations for refining future sampling efforts in order to maximize their total scientific benefits. Maximizing these benefits would improve the accuracy of stock assessments, and ultimately the quality of fishery regulations.

Although acquiring accurate representations of organismal abundances in the Gulf of Mexico’s marine ecosystem will always be a challenge, the present study revealed important conclusions unique to SEAMAP and CAGES:

1. SEAMAP would benefit from:
  - Concentrating sampling in near-shore and reef communities
  - Concentrating sampling during specific seasonal periods identified in Table 2
  - Expanding other FIM programs (e.g. Video Traps) to improve accuracy in specific species (e.g. Grouper and Red Snapper), as well as continuing sampling efforts using the protocols (pre 2009) of the SEAMAP Shrimp Trawl program
  - Focusing more effort on economically and ecological important species identified in Figs 2 through 4 (e.g. Grouper, Jacks, Mackerel, Pigfish, and Pompano)
2. CAGES would benefit from:
  - Concentrate sampling effort during seasonal periods identified in Table 4. Note: CAGES compared to SEAMAP had much clearer seasonal sampling periods per functional group/species

This analysis could be expanded in a follow-on study into a proper cost-benefit analysis of FIM effort by comparing the value of the data obtained from each FIM program against the operating costs of the FIM program. Such a study would require an assessment of fixed and variable costs (e.g. fuel, crew pay, etc…) through interviews and a search of government financial records.
